# On the Response to Hygrothermal Ageing of Fully Recyclable Flax and Glass Fibre Reinforced Polymer Composites

**DOI:** 10.3390/ma16175848

**Published:** 2023-08-26

**Authors:** Subrata Chandra Das, Chaman Srivastava, Stergios Goutianos, Angela Daniela La Rosa, Sotirios Grammatikos

**Affiliations:** ASEMlab—Laboratory of Advanced and Sustainable Engineering Materials, Group of Sustainable Composites, Department of Manufacturing and Civil Engineering, NTNU—Norwegian University of Science and Technology, 2815 Gjøvik, Norway or scdas.fibers@gmail.com (S.C.D.); stergios.goutianos@ntnu.no (S.G.);

**Keywords:** natural fibre composites (NFCs), flax fibre composites, GFRP, recyclable polymer matrices, bio-based epoxy, Elium^®^, thermosets and thermoplastics, durability, DMA (dynamic mechanical analysis), circular economy

## Abstract

The present work studies the response to hygrothermal ageing of natural fibre composites (NFCs) against synthetic fibre composites when using three different types of polymers as matrices. For ageing, coupons were fully immersed in distilled water at 23, 40, and 60 °C for a total ageing period of 56 days. Flax fibre-reinforced composites, using two recyclable polymer systems: (i) a bio-based recyclable epoxy and (ii) an acrylic-based liquid thermoplastic resin, were tested against conventional glass fibre-reinforced composites employing a synthetic (petroleum-based) epoxy. Different fibre/polymer matrix material combinations were tested to evaluate the effects of hygrothermal ageing degradation on the reinforcement, matrix, and fibre/matrix interface. The hygrothermal ageing response of unaged and aged composite coupons was assessed in terms of flexural and viscoelastic performance, physicochemical properties, and microscopy (SEM—Scanning Electron Microscopy).

## 1. Introduction

The usage of natural fibre composites (NFCs) is becoming popular in the composite industry due to certain environmental issues, in addition to other important strategic plans from regulatory bodies, such as the EU’s Green Deal and Circular Economy (CE) action plan in Europe [1,2]. Moreover, natural fibres possess significant advantages over synthetic glass fibres, including their low density, higher specific stiffness, low CO_2_ emissions during their life cycle period, and biodegradability [3,4,5]. Life Cycle Assessment (LCA) studies have revealed that NFCs have a lower environmental impact than GFRPs (Glass Fibre Reinforced Plastics), with similar polymer incorporation and application aspects [6,7]. Moreover, the employment of bio-based and/or recyclable polymer matrices promotes sustainability in composite products as well as compliance with the strategic CE action plan [1,2].

Flax is a plant-based natural fibre that is also commercially employed in the composite industry due to its availability in the form of various reinforcements and its superior mechanical performance compared with other plant fibres. The applications of flax fibre composites are found in the automotive, domestic, sports, and recreation sectors. Moreover, there is potential to use them in wind turbine blades as well as in aerospace and satellite structures [5,8]. Similar to other plant-based natural fibres, flax fibres are mainly composed of cellulose, hemicellulose, and pectin as the main chemical constituents and are therefore highly sensitive to moisture due to the presence of numerous -OH groups in their chemical structures. These numerous -OH groups attract and form H-bonds with water molecules. Moreover, flax fibres form a central hollow region that can reserve moisture within. Hence, significantly higher moisture absorption is observed in flax fibre composites, i.e., approximately 10–21 wt.% under room temperature conditions [9,10,11]. Water molecules penetrate inside the flax fibre composites via diffusion. As such, the fibres swell, plasticize, or soften, and the dimensions of the composites are also altered [11,12,13,14]. The pores, voids, and micro-cracks in the composite laminates are produced during manufacturing, and these also accelerate the rate of water uptake [12,14]. On the other hand, hydrophobic polymer matrices are found to be less compatible with hydrophilic flax fibres, so there is weaker fibre/matrix interfacial adhesion compared with synthetic fibres (glass, carbon, etc.), affecting the mechanical performance negatively. In humid environments, polymer resins also absorb moisture, swell, soften (or plasticize), and decompose (hydrolysis) as a function of prolonged exposure. The moisture absorbed inside the composites may cause swelling stresses that also generate micro-cracks in the matrix and thereby soften or plasticize the polymer matrix. Consequently, this results in interfacial deterioration, like debonding, which might lead to delaminations. The presence of inherent voids or micro-cracks may also enhance moisture penetration into the composite structure, which also contributes to interfacial deterioration. All these phenomena may cause degradation of the physico-mechanical properties of flax fibre composites [11,12,14,15,16]. It has also been reported that the coupling of high degrees of ageing temperature with moist environments may induce further acceleration of moisture uptake within the composite structure, accelerating further the overall degradation process [11,12,16,17,18,19]. However, due to additional post-curing, cross-linking, or enhanced crystallinity in the polymer matrix caused by accelerated ageing, mechanical properties may exhibit improvements for short periods [14,16,20,21]. Since glass fibres are hydrophobic, they demonstrate increased compatibility with polymers, thereby resulting in composites with improved resistance to hygrothermal environments (usually exhibiting moisture uptake of <1 wt.% at room temperature conditions [16,22]) compared to NFCs (exhibiting moisture uptake of 10–21 wt.% at similar conditions [9,10,11]).

With regards to polymers, a bio-based recyclable epoxy resin and an acrylic thermoplastic resin are used in this study due to their recyclability, and lower environmental impact without compromising performance or ease of processing compared to traditional non-recyclable petroleum-based epoxy resin systems [12,14,23,24,25,26].

The two recyclable polymer resin systems studied in this work have both made breakthroughs in the composites market due to their recyclability and processability. The bio-based recyclable epoxy is an “off-the-shelf” thermosetting polymer system, cured with “Recyclamine” [14], that is recyclable in facile chemical environments, allowing for both the polymer and the fibre reinforcement to be recovered. The studied acrylic-based liquid thermoplastic resin, under the trade name of Elium^®^ (Arkema, France), is a recyclable thermoplastic resin, processable with wet manufacturing methods such as vacuum-assisted infusion, which is the main processing method employed in the wind industry, among others [27,28]. Although the recyclability and processability of the two recyclable polymers have been studied, the long-term performance of these polymers, especially when combined with flax and other natural fibre reinforcements, has received little attention.

Thus, the present work aims to investigate the durability of flax fibre-reinforced recyclable polymer matrix composites, when subjected to hygrothermal ageing at 23, 40, and 60 °C for a total period of 56 days. The durability of flax composites was studied, employing two recyclable polymer matrices, and was contradicted with a petroleum-based epoxy as well as with traditional glass fibre-reinforced composites. In an attempt to reveal the individual effects of hygrothermal ageing, on the fibre reinforcement and polymer matrix systems, composite coupons possessing different fibre/matrix combinations were investigated. The performance of the aged composites was assessed and compared against un-aged ones (for reference purposes) in terms of water uptake, flexural properties, viscoelastic behaviour, physicochemical properties as well as surface damage caused by ageing, using Scanning Electron Microscopy (SEM). Since durability, in the long run, is a significant drawback of recyclable natural fibre composites being adopted by the industry, the goal of this work is to support the understanding of the behaviour of such recyclable composites when subjected to accelerated hygrothermal ageing environments.

## 2. Materials and Methods

### 2.1. Materials

Unidirectional (UD) flax fabric (FlaxDry UD 180, areal density 180 g/m^2^, fibre density 1.45 g/cm^3^) and UD glass fabric (areal density 220 g/m^2^, fibre density 2.5 g/cm^3^) used in this study were procured from EcoTechnilin, France, and Haufler Composites, Blaubeuren, Germany, respectively. Three types of matrix materials were employed: (i) a petroleum-based epoxy resin system (SP 106 resin and SP 106 slow hardener, mix ratio 100:18) supplied by Gurit, UK; (ii) a bio-based recyclable epoxy resin system (Polar Bear, with Recyclamine hardener, mix ratio 100:22) supplied by R*Concept, Spain; and (iii) an acrylic liquid thermoplastic resin (Elium^®^ 188 XO) by Arkema, France.

### 2.2. Methods

#### 2.2.1. Fabrication of Composites

**Figure 1:**
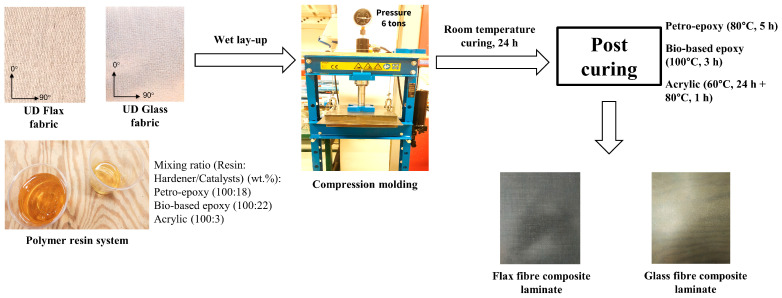
Composite manufacturing process [14].

The composite laminates were fabricated using a combination of wet lay-up and compression-moulding at room temperature according to our previous study [14] and are exhibited in Figure 1. After 24 h of curing, the cured (cold-pressed) laminates were post-cured, according to the respective datasheets: 80 °C × 5 h, for the petro-based epoxy composites, 100 °C × 3 h for the bio-based epoxy resin-based composites, and 60 °C × 24 h +80 °C × 1 h for the acrylic-based composites. The fabricated composite laminates were (i) flax/petro-epoxy (FFRP1), (ii) flax/bio-based recyclable epoxy (FFRP2), (iii) flax/acrylic thermoplastic (FFRP3), (iv) glass/petro-epoxy (GFRP1), (v) glass/bio-based recyclable epoxy (GFRP2), and (vi) glass/acrylic thermoplastic (GFRP3). After post-curing, the fabricated laminates were machined into coupons at the desired dimension. The physical properties of the fabricated composite laminates are presented in Table 1.

#### 2.2.2. Hygrothermal Ageing

Hygrothermal ageing of the studied composites was conducted in water tanks using distilled water at 23, 40, and 60 °C ageing temperatures. Ageing was carried out for a total of 56 days, with testing intervals at 7, 14, 28, and 56 days. The total ageing period of 56 days was selected as the required time to reach the saturation level of flax fibre composites (FFRPs) test coupons (specimens) to degrade at the selected hygrothermal ageing environments, which is in agreement with previous works in literature [13,29,30,31].

#### 2.2.3. Testing

##### Flexural Testing

The flexural tests in a three-point bending clamp of the unaged and aged composite specimens after hygrothermal ageing under different conditions were performed according to the ISO 14125 standard [32] by using a Universal Testing Machine (Instron 5966, 10 kN load cell) with a crosshead speed of 2 mm/min at a span distance of 80 mm. The dimensions of the flexural test specimens were 100 (l) × 15 (w) mm^2^. At least five coupons were tested for each case. The flexural strength and flexural modulus were calculated using the following formulas:

Flexural strength,

(1) $$\sigma_{f}=\frac{3PL}{2bd^{2}}$$

Flexural modulus,

(2) $$E=\frac{L^{3}m}{4bd^{3}}$$

where,

P = the maximum applied load,

L = the length of support span,

m = the slope of the tangent,

b = the width of the specimen, and

d = the thickness of the specimen.

##### Dynamic Mechanical Analysis (DMA)

The DMA testing of the unaged and aged composite specimens after hygrothermal ageing was performed according to ASTM D 5023 using a dynamic mechanical analyzer (DMA 850, Discovery, USA). Coupons were machined in dimensions of 64 (l) × 13 (w) mm^2^. For the test, a temperature rate of 3 °C/min was selected, from 30 to 140 °C at 1.0 Hz frequency and amplitude of 30.0 µm. The resulting storage modulus (E’) was calculated from the E’ vs. temperature curves at 30 °C, and the glass transition temperature (T_g_) was calculated from the peak of tan δ curves. At least four coupons were tested for each case.

Moisture uptake is a sample-dimension-dependent problem, meaning that different sample sizes will result in different moisture diffusivity and amount of moisture uptaken [33]. Mechanical and viscoelastic testing has been conducted herein to evaluate, as a direct comparison, coupons aged for the same amount of time. Obviously, same-sized GFRP coupons absorb less moisture as compared to FFRPs for the same amount of time, or else FFRPs reach moisture saturation at a significantly higher rate.

##### Fourier Transform Infrared (FTIR) Spectroscopy

To compare the changes in the chemical signature as a function of hygrothermal ageing, the FTIR spectra of the unaged and 56-day-aged composite coupons were scanned using an FTIR-ATR (Attenuated Total Reflection) (Perkin Elmer^®^).

##### Scanning Electron Microscopy (SEM)

SEM micrographs of the surface of the unaged and aged composite specimens after 56 days of hygrothermal ageing under different conditions (23, 40, and 60 °C) were collected using an SEM facility equipped with a field emission electron gun (Zeiss-Supra 55VP-FEG-SEM).

## 3. Results and Discussions

### 3.1. Flexural Properties

**Figure 2:**
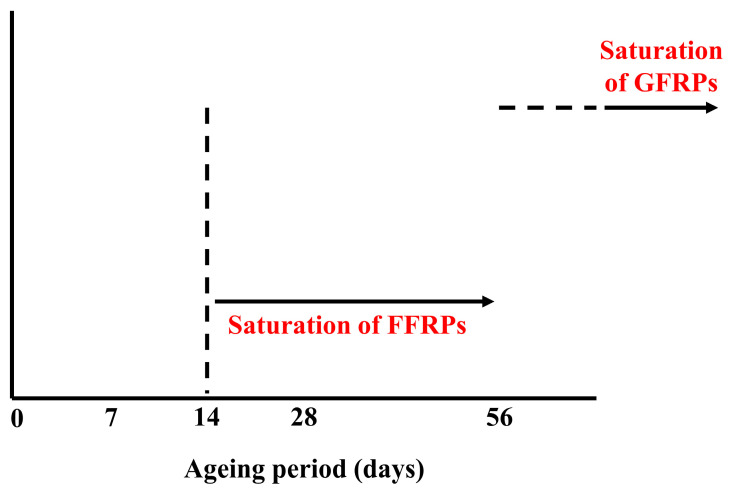
Saturation of flax fibre composites (FFRPs) and glass fibre composites (GFRPs) in hygrothermal ageing environments.

The moisture uptake (%) of flexural test specimens during different ageing periods is tabulated in Table 2 for flax fibre composites (FFRP1, FFRP2, and FFRP3) and glass fibre composites (GFRP1, GFRP2, and GFRP3). It is seen from the moisture uptake (%) values that the flax fibre composites (FFRPs) reached the saturation level approximately after 14 days of the ageing period in hygrothermal environments, which is also depicted in Figure 2. On the other hand, the moisture uptake values of glass fibre composites are significantly lower, which also require a substantially longer period to reach saturation level (Figure 2) due to the hydrophobic nature of glass fibres and polymer matrices. These phenomena can be reflected in the degradation behaviours of FFRPs and GFRPs in hygrothermal ageing conditions.

**Figure 3:**
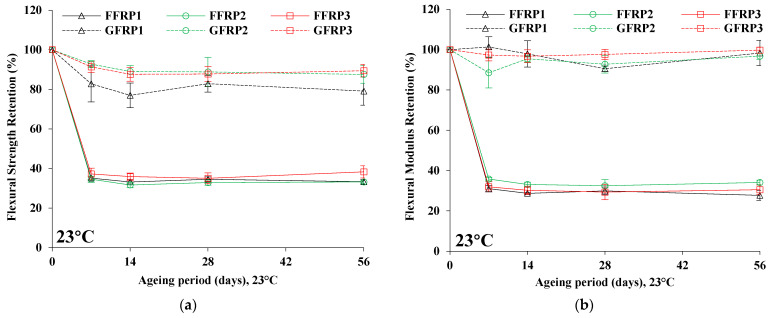

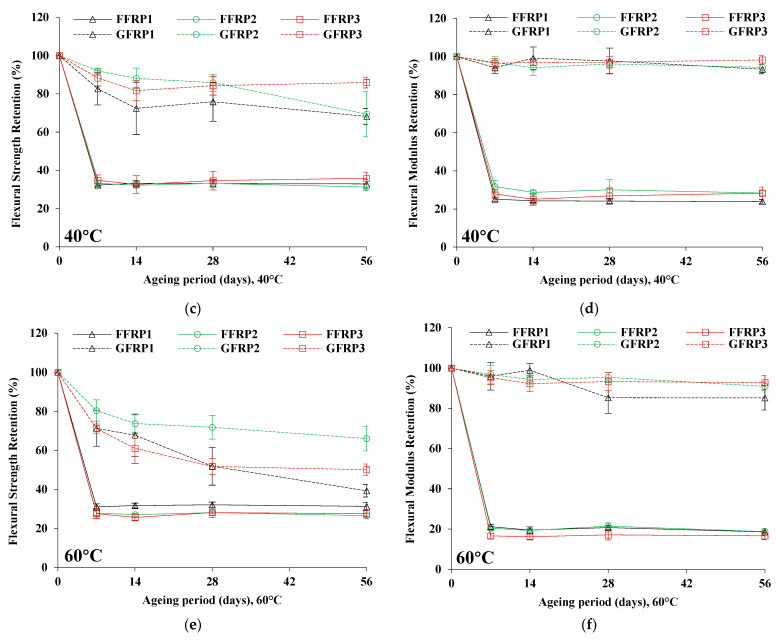
Retention of flexural strength at 23 °C (**a**), 40 °C (**c**), and 60 °C (**e**), as well as the retention of the flexural modulus at 23 °C (**b**), 40 °C (**d**), and 60 °C (**f**).

Table 3 and Table 4 tabulate the flexural properties of flax (FFRP1, FFRP2, and FFRP3) and glass (GFRP1, GFRP2, and GFRP3) fibre-reinforced polymer composites subjected to three different hygrothermal ageing environments, respectively. Figure 3 depicts the property retention (using Property Retention (%) = P_dayx_ − P_day0_/_Pday0_) of flexural strength and flexural modulus after hygrothermal ageing for 7, 14, 28, and 56 days for the three hygrothermal ageing environments: 23, 40, and 60 °C. For the case of flax composites, as can be seen, after 7 days of ageing and for all ageing conditions (23, 40, and 60 °C), significant degradation of flexural properties is observed. After that, flexural properties oscillate around a constant value without exhibiting any further loss in flexural performance for the rest of the ageing period (up to 56 days).

More specifically, in terms of flexural strength, after 7 days of ageing at 23 °C, the petro- and bio-based epoxy/flax composites (FFRP1 and FFRP2) revealed almost identical (approximately 65%) extents of degradation, while FFRP3 degraded to 63%. Similarly, after 7 days of ageing at 23 °C, the decline in flexural modulus was similar for FFRP1 and FFRP2, while FFRP2 showed slightly lower values. Thereafter, from 14 to 56 days of ageing time, the flexural properties of the flax composites were not significantly affected. After 56 days of ageing, FFRP1 and FFRP2 were found to have the same magnitude of reduction in terms of flexural strength (67%), while FFRP3 degraded to 62% of the original state. In terms of modulus, FFRP1 and FFRP3 values lowered to 70–72%, while FFRP2 lowered to 66%, compared to the initial value. For all flax composite cases, the magnitude of flexural property loss was higher for hygrothermal ageing at 40 and 60 °C. After 56 days of ageing at 40 °C, flexural strength reduced to approximately 67, 69, and 64% for FFRP1, FFRP2, and FFRP3, respectively, while flexural modulus dropped to approximately 72% for both FFRP2 and FFRP3 and 76% for FFRP1. On the other hand, 60 °C hygrothermal ageing caused a slightly greater loss of flexural strength values, which was almost similar for FFRP2 and FFRP3 (72–73%) and 69% for FFRP1, whilst the degradation of flexural modulus was almost similar for all cases (ranging between 81 and 83%).

Ageing at 23 °C caused drops of 62–67% and 66–72% in flexural strength and modulus, respectively, for FFRP composites after 56 days of ageing, which is significantly higher than in the study of Yan and Chouw [10] and comparatively lower than what is reported by Santosh et al. [34]. The rate of degradation in hygrothermal conditions may be dependent on the interfacial adhesion between reinforcement and polymer matrix, ageing environments, duration of ageing, and the properties of the employed reinforcement and matrix.

Kollia et al. [12] reported approximately 36–43% and 45–53% drop in flexural strength in 40 and 60 °C hygrothermal ageing, respectively, of woven flax/bio-epoxy composites to the point of saturation, whilst approximately 26–55% and 50–64% drop occurred, respectively, in the flexural modulus under the same ageing conditions. In another work, Dhakal et al. [35] reported a drop of approximately 40 and 69% in the flexural strength and flexural modulus, respectively, for flax/bio-epoxy composites in a 25 °C water medium after 961 h (~40 days). Higher degradation in the flexural strength and modulus is observed in the current study than in the study of Kollia et al. [12]. On the other hand, low flexural strength degradation and similar flexural modulus degradation were reported by Dhakal et al. [35] (but not in the current study of FFRP composites at room temperature conditions.

Given the above, it can be postulated that the incorporation of different polymer matrices has a negligible effect on the flexural performance after hygrothermal ageing of FFRPs, as performance is dominated by the hydrophilicity of flax fibre reinforcement.

No clear trend in the degradation of flexural strength and modulus is observed for glass fibre composites for the tested ageing periods and temperatures, as depicted in Figure 3. In 23 °C ageing environments, the degradation of flexural strength was more pronounced for petro-based epoxy composites (GFRP1) (21%), while the bio-based epoxy composites (GFRP2) and acrylic thermoplastic-based composites (GFRP3) exhibited less degradation in flexural strength (11–12%) at 23 °C and after a 56-day ageing period. Conversely, a slight drop in the flexural modulus was evident for GFRP1 and GFRP2 (2–3%), but a negligible property change was exhibited by GFRP3 after 56 days of ageing at 23 °C.

For the cases of ageing at 40 and 60 °C, more pronounced degradation in terms of flexural strength values was observed. As such, for ageing at 40 °C, approximately a 32% drop in flexural strength for both GFRP1 and GFRP2, was calculated after 56 days, and significantly less (14%) for GFRP3. At 60 °C after 56 days, a significant drop in flexural strength occurred for all glass fibre composites, with 61, 34, and 50% for GFRP1, GFRP2, and GFRP3, respectively. When comparing all three glass composites, GFRP3 exhibited higher retention of flexural properties than both epoxy polymer-based GFRPs, which may be attributed to the interfacial adhesion between the glass fibres and acrylic resin matrix. Almost identical degradation behaviour was observed for both petro- and bio-based epoxy/glass composites (GFRP1 and GFRP2) aged at 23 and 40 °C. However, for ageing at 60 °C, the degradation in flexural properties was comparatively higher for GFRP1 than for GFRP2, which was not expected due to the bio-based content of the matrix forming GFPR2. In terms of flexural modulus, a similar behaviour was obtained for GFRP1 and GFRP2, with GFRP1 revealing a more pronounced drop (15%) for ageing at 60 °C.

Kollia et al. [36] reported a decrease of approximately 26 and 32% in flexural strength for woven glass fibre-reinforced cyanate ester composites after 61 days of ageing under 40 and 60 °C hygrothermal conditions, respectively. The present study, with similar conditions to Guo et al., shows a significantly higher degradation in flexural strength for the petro- and bio-based epoxy GFRPs [37]. Moreover, at 40 °C, there is a slightly higher degradation of both GFRP1 and GFRP2 than in glass/cyanate ester composites reported by Kollia et al. [36]. However, in this study, GFRP1 shows an almost twofold higher degradation in the flexural strength values at 60 °C than found in [36], while GFRP2 exhibits almost similar values of degradation in the flexural strength with [36].

Agreement in the flexural behaviour of GFRP3 was found with the work reported by Nash et al. [38], which studied the behaviour of UD glass/acrylic liquid thermoplastic (namely Elium 150) composites at 35 °C hygrothermal ageing for 28 days and revealed a 17.3 and 0.4% loss of flexural strength and modulus, respectively. On the other hand, Bandaru et al. [39] revealed an approximately 59 and 62% decline in the flexural strength and modulus, respectively, after 60 days of hygrothermal ageing at 60 °C for the non-crimp quadriaxial glass fibre-reinforced acrylic (Elium 150) thermoplastic composites. Here, the drops in flexural strength and modulus were 9 and 55% higher, respectively, than in GFRP3 composites that were aged at 60 °C for 56 days in the current work. This may be due to the strong interfacial adhesion between glass fibres and acrylic resin and the UD structure of the glass reinforcement in GFRP3 composites.

### 3.2. DMA

The moisture uptake (%) of DMA test specimens during different ageing periods is tabulated in Table 5 for flax fibre composites (FFRP1, FFRP2, and FFRP3) and glass fibre composites (GFRP1, GFRP2, and GFRP3). Similar to the flexural test specimens, the DMA test specimens exhibited similar moisture uptake and saturation behaviour under hygrothermal ageing environments (Figure 2).

#### 3.2.1. Storage Modulus (E’)

Table 6 documents the storage modulus (E’) of unaged and aged FFRP and GFRP composites under the different hygrothermal ageing conditions of the study for all testing intervals. As aforementioned, DMA tests were conducted in three-point bending mode. The values of E’ of the unaged composites were calculated to be 21.99, 20.96, 19.24, 37.84, 34.02, and 33.44 MPa for FFRP1, FFRP2, FFRP3, GFRP1, GFRP2, and GFRP3, respectively.

**Figure 4:**
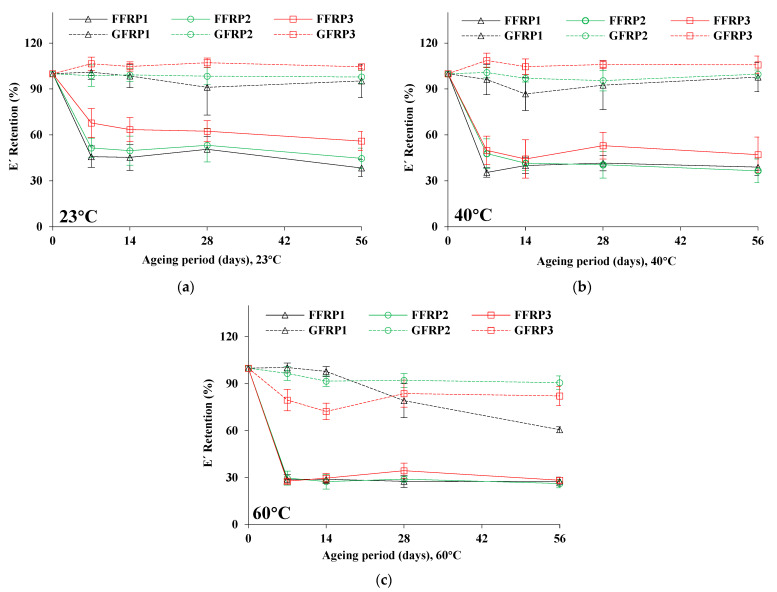
Retention of E’ as a function of hygrothermal ageing under different conditions: (**a**) 23 °C, (**b**) 40 °C, and (**c**) 60 °C.

Figure 4 shows the retention of E’, as a function of hygrothermal ageing at 23, 40, and 60 °C. In the case of FFRPs, the values of E’ dropped significantly after 7 days of ageing and for all ageing conditions, which confirms the flexural modulus performance (see Figure 3b,d,f). After that time interval, no significant change in E’ values was observed for ageing up to 56 days or for all ageing conditions. Nevertheless, higher temperature hygrothermal ageing, induced a higher degradation of E’ values, as expected [40,41]. Interestingly, E’ values increased for GFRP3, which may be attributed to the effect of additional crosslinking, or the enhancement of crystallinity in the polymer matrix system(s) [16,20,21].

It was also observed that both FFRP1 and FFRP2 exhibited almost similar lowering in E’ values, whilst E’ values for ageing at 23 and 40 °C, resulted in comparatively lower degradation for FFRP3 coupons. This may be due to comparatively lower damage in the interfacial adhesion in the case of FFRP3 than that of epoxy polymer-based composites (FFRP1 and FFRP2) in these ageing conditions (23 and 40 °C). However, for ageing at 60 °C, all three FFRP1, FFRP2, and FFRP3 exhibited a similar decline in E’ values (72–74% drop), as illustrated in Figure 4c.

In the case of GFRPs, the changes in E’ values were marginal for ageing at 23 °C hygrothermal ageing conditions, resulting in 5 and 2% E’ value reduction after 56 days of ageing for the case of GFRP1 and GFRP2, respectively, while at the same time E’ values increased for GFRP3 by 8%. On the contrary, for ageing at 40 °C, the E’ values underwent a 2% decline for GFRP1, while no change occurred for GFRP2 and a 10% increase was recorded for the GFRP3 composites. The slight decrease in E’ values as a function of hygrothermal ageing at 23 and 40 °C, indicates increased ageing resistance for all cases of GFRPs [16,42].

However, the degradation of E’ was more pronounced for all GFRPs at 60 °C. That said, GFRP1 resulted in a 39% lowering of E’ values, while 4 times lower degradation in E’ values were calculated for GFRP2 than for GFRP1. Hence, the bio-based epoxy GFRPs performed better than petro-based GFRPs, in terms of E’ for ageing at high temperatures, which was also the case for flexural ageing (see Figure 3, Section 3.1). On the other hand, the degradation (18%) in E’ values was twice as high for GFRP3 compared with GFRP2. In summary, the magnitude of degradation at 60 °C follows the order GFRP1 (39%) > GFRP3 (15%) > GFRP2 (9%).

Given the above, it can be concluded that storage modulus (E’) is highly dependent on the type and properties of reinforcing fibres and very marginally on the type of matrix, dominating any stiffness changes caused by environmental exposure.

#### 3.2.2. Glass Transition Temperature (T_g_)

**Figure 5:**
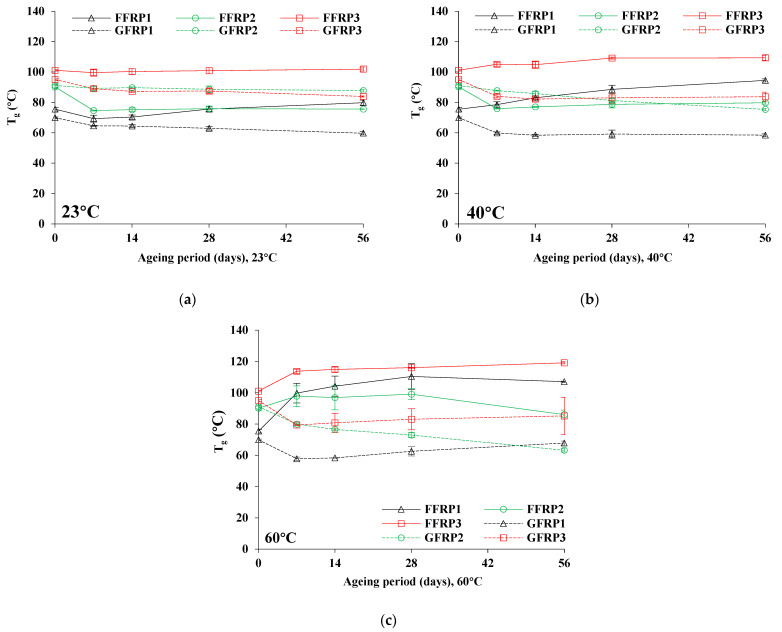
Changes in T_g_ (°C) as a function of hygrothermal ageing under different conditions: (**a**) 23 °C, (**b**) 40 °C, and (**c**) 60 °C.

Table 7 tabulates the T_g_ values of unaged and aged flax and glass fibre composites after ageing at the three ageing conditions (23, 40, and 60 °C) for 7, 14, 28, and 56 days. Figure 5 depicts the changes in T_g_ (°C) values as a function of hygrothermal ageing at 23, 40, and 60 °C.

As can be seen from Figure 5a, the T_g_ values for FFRP1 show a marginal oscillation around the original (unaged) value after ageing at 23 °C for a total period of 56 days. However, T_g_ values changed significantly due to ageing at 40 and 60 °C, with T_g_ reaching a peak value at 56 days for ageing at 40 °C and 28 days for ageing at 60 °C.

The increase in T_g_ values determined for FFRP1 after hygrothermal ageing may be attributed to both the additional cross-linking caused by the effect of thermal energy and, especially for epoxies, the phenomenon is likely to be enhanced by the effect of bound water, which causes new (weaker) H-bonds [43,44], establishing a secondary network of crosslinking within the polymer matrix [20]. Moreover, the existence of different functional groups, coupling agents, and additives, usually present in commercial polymer resins, maybe one more reason for higher recorded T_g_ values, as well as the increased height of secondary peaks in the tan δ curves. Lastly, anti-plasticization or embrittlement phenomena as well as extremely rapid evaporation of moisture from the structure of the coupons during the heating process of DMA testing [45] may also contribute to the increase in T_g_.

In the case of FFRP2, T_g_ values decreased by 16% after 7 days of ageing at 23 °C, while drops in T_g_ values by 12% and 5% were recorded for ageing at 40 and 60 °C, respectively, after 56 days. The values of T_g_ increased for 28 days of ageing at 60 °C, which may be attributed to possible post-curing, crosslinking, or increased crystallinity effects of the polymer [14,16,20,21]. In the case of FFRP3, T_g_ values remained almost intact for ageing at 23 °C. However, for ageing at 40 °C, T_g_ values increased marginally (by 4–8% after 56 days), which may be due to the polymer post-curing effect [16,20,21], whereas for ageing at 60 °C the increase in T_g_ values was more pronounced (13–18% increase), exhibiting similar behaviour to FFRP1 [14,20,43,44,45].

For the case of GFPRs, after 56 days of ageing, the values of T_g_ for GFRP1, GFRP2, and GFRP3 were calculated to change by 15%, 4%, and 12% for 23 °C; 16%, 17%, and 12% for 40 °C; and 3%, 31%, and 14% for ageing at 60 °C, respectively.

In the case of GFRP1, the T_g_ values dropped by approximately 16% after 56 days of ageing at 23 °C and 40 °C. However, only a 3% decrease in T_g_ was recorded for ageing at 60 °C, which could be attributed to the likelihood of increased additional crosslinking [16,20,21]. In the case of GFRP2, T_g_ values changed slightly at 23 °C, while the degradation degree increased for ageing at 40 and 60 °C, revealing a decline of 17% for ageing at 40 °C, and 31% for ageing at 60 °C. For GFRP3, a decrease by 12–14% of T_g_ values was observed after 56 days of ageing, and for all three ageing temperatures.

### 3.3. Fourier Transform Infrared (FTIR) Spectroscopy

**Figure 6:**
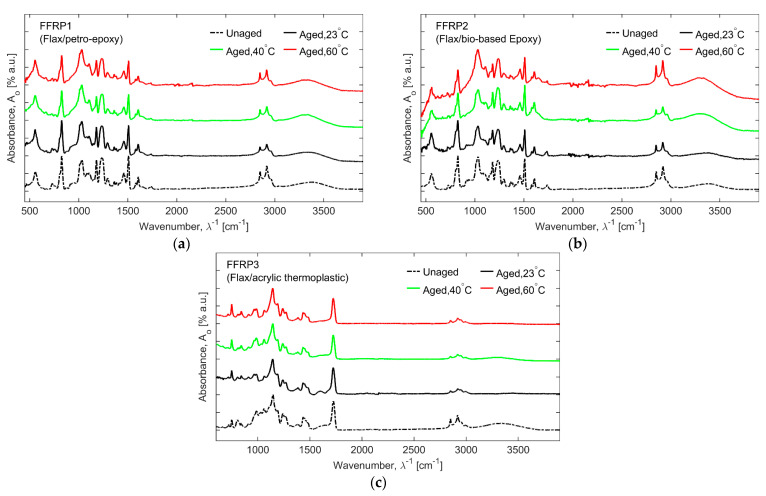
FTIR spectra of unaged and aged flax fibre composites after 56 days of hygrothermal ageing at 23, 40, and 60 °C baths: (**a**) FFRP1, (**b**) FFRP2, and (**c**) FFRP3.

**Figure 7:**
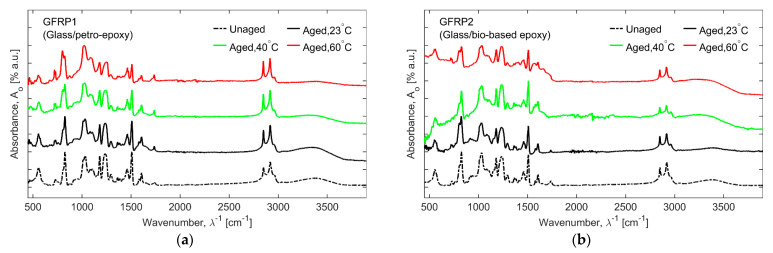

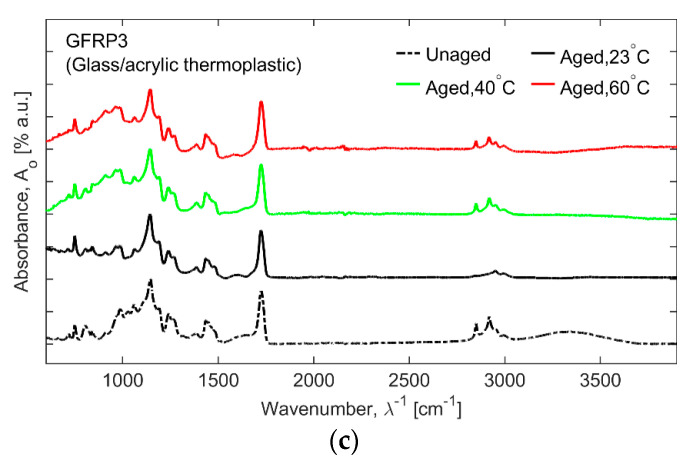
FTIR spectra of unaged and aged glass fibre composites after 56 days of hygrothermal ageing at 23, 40, and 60 °C baths: (**a**) GFRP1, (**b**) GFRP2, and (**c**) GFRP3.

Figure 6 and Figure 7 present the FTIR signals in the fingerprint and group frequencies regions for flax fibre composites (FFRP1, FFRP2, and FFRP3) and glass fibre composites (GFRP1, GFRP2, and GFRP3), respectively, for unaged and 56-day hygrothermally aged test coupons at 23, 40, and 60 °C. To compare and quantitively analyze the FTIR spectra, data was normalized against the maximum values and then compared in the progression of the ageing temperature.

**Figure 8:**
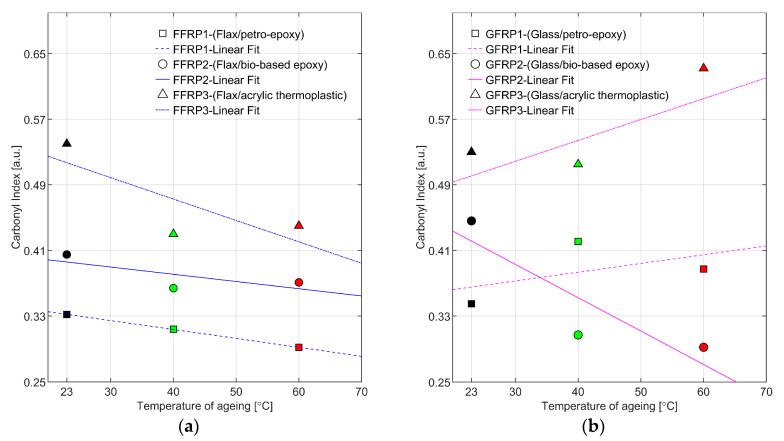
Carbonyl index as a function of 56 days hygrothermal ageing at 23, 40, and 60 °C baths for flax fibre composites (**a**) and glass fibre composites (**b**).

As can be seen from Figure 6 and Figure 7, there was no new characteristic peak observed as a function of hygrothermal ageing. It can be observed that the FTIR spectra for both the petro- and bio-based epoxies had a similar pattern. The common peaks recorded are at 3370 cm^−1^ for O-H stretching, at 1606 cm^−1^ for stretching of C=C aromatic ring, at 1507 cm^−1^ for stretching of aromatic C-C, at 1461 cm^−1^ for C-H bending of aliphatic groups, at 1234 cm^−1^ for stretching of aromatic ether, at 1032 cm^−1^ for stretching of the C-O-C ethers, and at 825 cm^−1^ for aromatic C-H plane deformation [41,46]. As a general trend, the peak heights for the petro- and bio-based epoxies decreased as the ageing temperatures and periods increased. The -OH group absorption is a sign of hydrolysis reaction, and in FFRP1 and FFRP2, the absorption increases, which is contrary to GFRP1 and GFRP2, where the change was negligible. This can be attributed to the hygroscopic nature of natural fibre reinforcements like flax [41,46]. The degradation behaviour of flexural properties (see Section 3.1) of FFRPs and GFRPs can be approximated with the carbonyl peak height to assess the levels of degradation, as presented in Figure 8. It is observed that, in the case of flax fibre-reinforced composites, the carbonyl peak height decreases as the ageing temperature increases for the ageing duration of 56 days. This is synonymous with degradation trends observed, for example, in flexural testing of FFRP. The behaviour can be attributed to the combined degradation of hemicellulosic fibrous molecules inside flax and the carbonyl conversion inside the matrix. It has to be noted that though the carbonyl is water-soluble, the exact presence could vary [47].

In the case of glass fibre, the carbonyl peak increases instead of the bio-based epoxy polymer. It is also observed during the mechanical testing that GFRP-based composites undergo minimal degradation as well.

In the case of acrylic thermoplastic (Figure 6c and Figure 7c), a very distinct FTIR curve is observed, where the reference peak is at 1723 cm^−1^ for the -C=O peak from the methacrylate functional group, at 1140 cm^−1^ is associated with the C–O–C bond and at 1450 cm^−1^ for bending vibration of the C-H group. Negligible changes observed are in the carbonyl peak, where the peak height decreases for FFRP composites and seems to increase for GFRP composites. The change in the peak height can be hypothesized with the degradation by hydrolysis.

### 3.4. Surface Damage (SEM)

**Figure 9:**
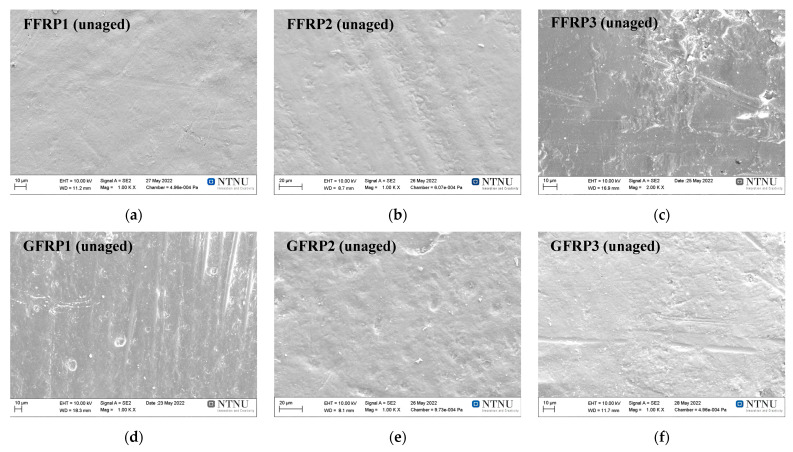
SEM images of the surfaces of unaged flax fibre composites: FFRP1 (**a**), FFRP2 (**b**), FFRP3 (**c**), and unaged glass fibre composites: GFRP1 (**d**), GFRP2 (**e**), GFRP3 (**f**) [14].

**Figure 10:**
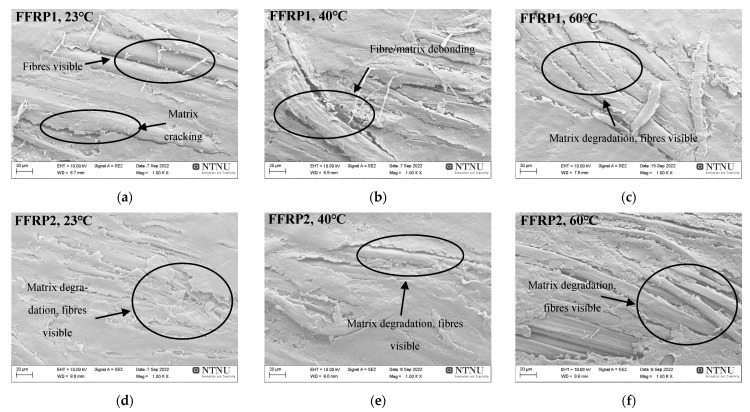

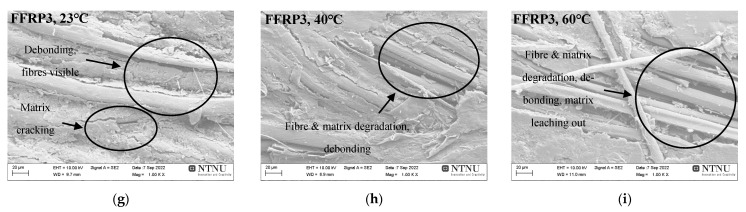
SEM images of the surfaces of aged FFRP1 (**a**–**c**), FFRP2 (**d**–**f**), and FFRP3 (**g**–**i**) after 56 days of hygrothermal ageing at 23, 40, and 60 °C.

**Figure 11:**
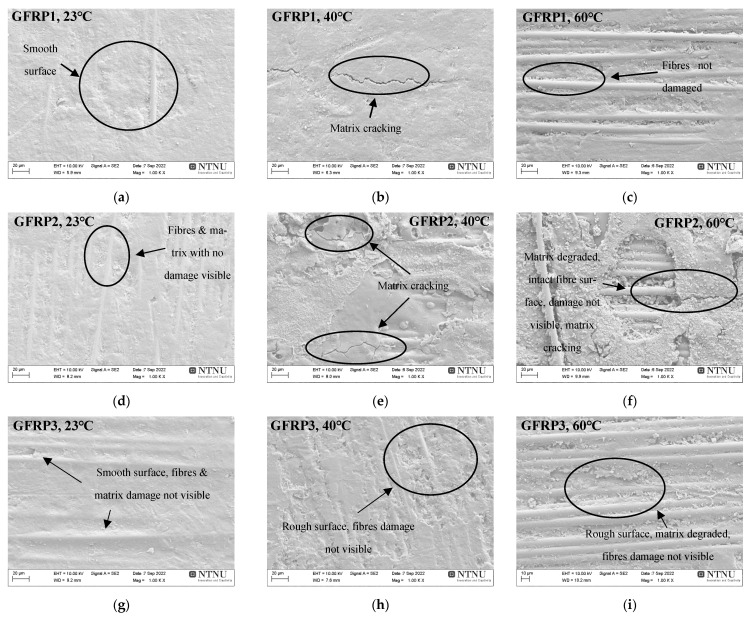
SEM images of the surfaces of aged GFRP1 (**a**–**c**), GFRP2 (**d**–**f**), and GFRP3 (**g**–**i**) after 56 days of hygrothermal ageing at 23, 40, and 60 °C.

SEM analysis was performed to scrutinise any induced changes in the morphology of the surface of hygrothermally aged coupons for 56 days in all three ageing environments. For reference, micrographs were compared with micrographs recorded on unaged coupons. Thus, Figure 9 presents SEM micrographs of the unaged (reference) FFRP and GFRP composite coupons, while Figure 10 and Figure 11 depict the scanned micrographs for the aged FFRP and GFRP coupons, respectively. In the case of unaged coupons (Figure 9), apart from manufacturing-induced marks, micrographs showed no evidence of damage or the presence of voids or cracks.

In the case of the aged FFRP1, FFRP2, and FFRP3 coupons, significant surface changes can be observed in Figure 10, as compared to the unaged cases of Figure 9. Ageing has eroded the top layer of the polymer, creating crazing, increasing the surface roughness, and in this way exposing the reinforcing fibres to environmental exposure, causing matrix cracking, fibre/matrix debonding, etc. [10,14,41,48].

Similarly, matrix cracking, surface roughness, and signs of matrix deterioration are visible in the case of aged GFRPs, as seen in Figure 11. No indication of fibre damage was visible due to the increased glass fibre resistance to ageing. Furthermore, as can be seen in Figure 11, elevated temperatures of ageing induce brittle damage to the surface of GFRPs [49], causing matrix micro-cracking and debonding without affecting the surface of glass fibres [14,22,36].

### 3.5. Flax Composites vs. Glass Composites

**Figure 12:**
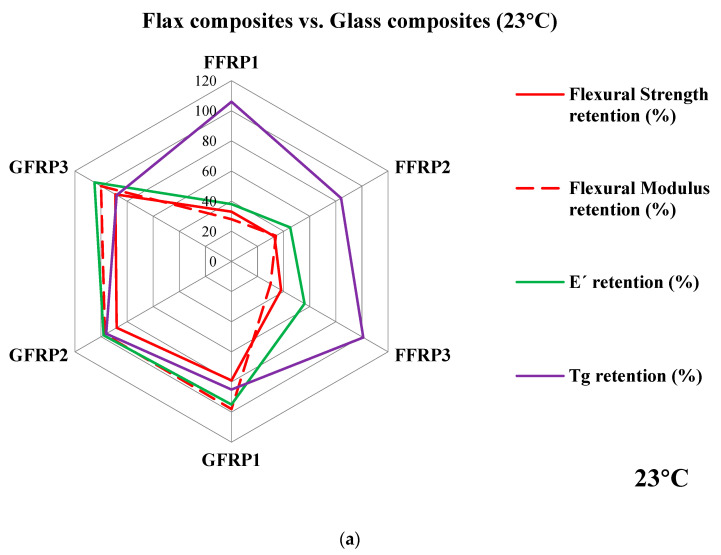

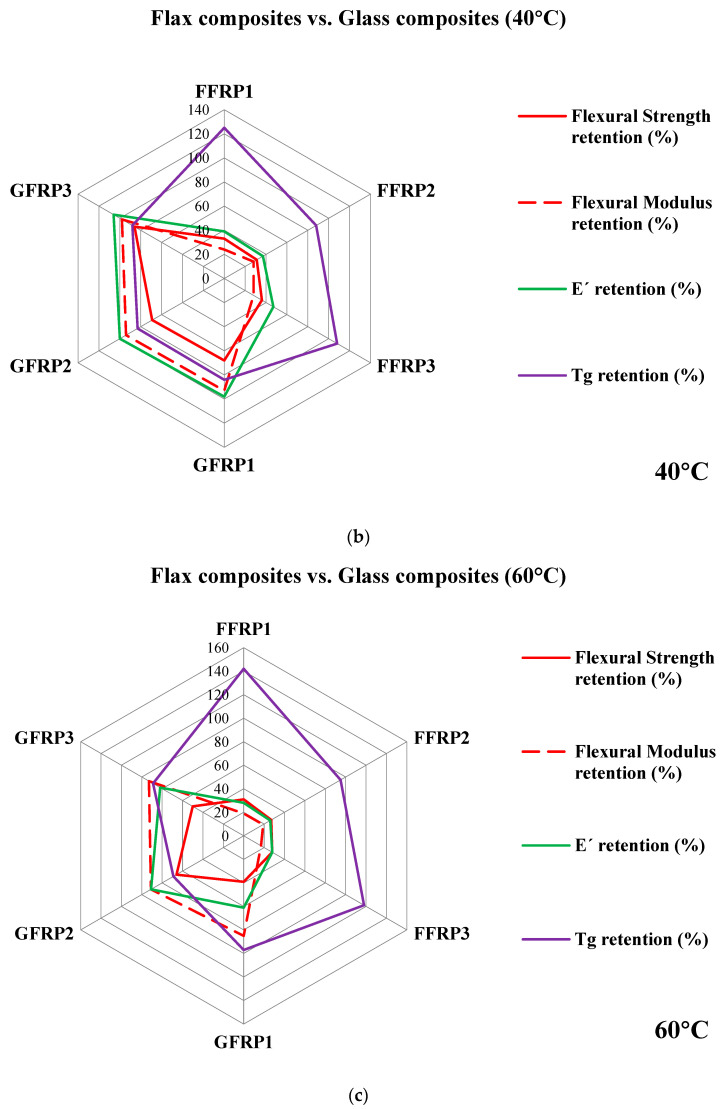
The comparative properties of flax composites and glass composites after 56 days of hygrothermal ageing under different conditions: (**a**) 23 °C, (**b**) 40 °C, and (**c**) 60 °C.

Figure 12a–c illustrates the comparative flexural and viscoelastic properties based on the retention (%) values as a function of hygrothermal ageing for 56 days at 23, 40, and 60 °C, for the studied composite cases FFRP1, FFRP2, FFRP3, GFRP1, GFRP2, and GFRP3. As shown in Figure 12, the three types of GFRP composite laminates exhibited significantly higher values of retention in terms of flexural and viscoelastic performance after hygrothermal ageing, which is mainly due to the increased hydrophilic character of GFRP composites [10,14,50]. Therefore, it can be postulated that reinforcing fibres play a dominant role in the long-term performance of natural fibre composites when exposed to hygrothermal ageing environments. In the case of FFRP composites, it was observed that for 23 and 40 °C hygrothermal ageing, the acrylic thermoplastic-based flax composite (FFRP3) performed well compared to the petro- and bio-based epoxy/flax composites (FFRP1 and FFRP2). However, in the case of ageing at 60 °C, all three FFRP composite cases exhibited almost similar extent of degradation in their flexural properties, denoting that above room temperature ageing conditions, ageing degradation is mainly driven by the type of fibre reinforcement. Overall, FFRP3 exhibited adequate performance after ageing at 23 and 40 °C environments, while FFRP2 exhibited almost similar, or in some cases, greater performance than the FFRP1 type.

In the case of GFRPs, the GFRP3 type exhibited increased retention of mechanical and viscoelastic performance as a response to hygrothermal ageing and for all environments. This may be due to the establishment of adequate fibre-matrix interfacial adhesion between the acrylic resin and glass fibres. Thus, it could be postulated that among all GFRP types, GFRP3 exhibited superior behaviour. However, it is worth mentioning that bio-based epoxy GFRPs (GFRP2) perform better than their petro-based counterparts (GFRP1). Hence, considering the overall performance, it can be stated that the superiority of behaviour follows the order GFRP3 > GFRP2 > GFRP1.

## 4. Conclusions

In this work, we studied the performance of flax fibre composites and glass fibre composites while using three different types of polymers as matrices when aged at three different hygrothermal ageing environments, at 23, 40, and 60 °C, for a total of 56 days. Different fibre/polymer matrix material combinations were tested to evaluate the effects of hygrothermal ageing degradation on the reinforcement, matrix, and fibre/matrix interface. The hygrothermal ageing performance of unaged and aged composite coupons was assessed via water uptake, flexural and viscoelastic behaviour, physicochemical changes, and surface analysis using SEM micrographs. The main findings of this work are as follows:

- Flax fibre composites demonstrate noticeably higher moisture uptake values than their glass fibre counterparts when using the same polymer matrix. Therefore, moisture uptake behaviour is predominantly dependent on the type of reinforcement rather than the type and nature of the polymer matrix. In addition, the ageing temperature has a significant influence on the moisture uptake behaviour.. Higher ageing temperature accelerates the rate of moisture uptake and reduces the time to reach moisture saturation.
- Hygrothermal ageing induced more significant damage to FFRP than GFRP composites when studying flexural and viscoelastic performance. In the case of flax fibre composites, significant flexural property degradation occurred after the first 7 days of ageing with barely noticeable changes for ageing up to 56 days. Higher ageing temperatures induced slightly higher degradation of the flexural properties.
- In the case of polymers, an almost similar degradation behaviour was reported for all the flax composites. On the other hand, acrylic thermoplastic-based glass composites (GFRP3) performed better than the petro- and bio-based glass composites (GFRP1 and GFRP2) after ageing at 23 and 40 °C, while GFRP2 performed well for ageing at 60 °C. GFRP2 (bio-based epoxy) exhibited comparable or in some cases even better performance than GFRP1 (petro-based) composite laminates.
- The retention of storage modulus (E’) values, exhibited almost identical behaviour to flexural modulus retention values for all unaged and aged coupons tested in this study. The T_g_ was influenced by the effect of hygrothermal ageing, while the effect was more pronounced for the case of FFRPs and high ageing temperatures (40 and 60 °C). With regards to the type of polymer matrix, T_g_ values changed with the following: FFRP3 > FFRP1 > FFRP2 at 23 °C, FFRP3 > FFRP2 > FFRP1 at 40 °C, and FFRP2 > FFRP3 > FFRP1 at 60 °C. In the case of GFRPs, the retention of T_g_ values followed the order: GFRP2 > GFRP3 > GFRP1 at 23 °C, GRP3 > GFRP2 > GFRP1 at 40 °C, and GFRP1 > GFRP3 > GFRP2 at 60 °C.
- From FTIR testing, no new characteristic peaks were observed as a function of hygrothermal ageing; rather, the intensity was lowered, which indicates the hydrolysis of the exposed materials, i.e., physical changes occurred by hygrothermal ageing. The effect was more severe in the case of FFRPs and at high-temperature ageing.
- The SEM observations revealed noticeable surface damage (matrix micro-cracking, fibre damage) that was more pronounced in the case of flax fibre composites than the glass fibre counterparts. Due to the hydrophilic nature of flax fibres, they are more susceptible to erosion and degradation induced by moisture.

In summary, it was evident that the recyclable bio-based epoxy and the acrylic liquid thermoplastic-based composites exhibited similar, and in some cases, greater performance than the tested petroleum-based epoxy composites, after hygrothermal ageing, when considering flexural and viscoelastic property changes. This may be promising proof that the studied recyclable polymers may be good candidates to replace petroleum-based polymers, in future composite engineering applications. In addition, as was pointed out, the performance of composites at high-temperature hygrothermal ageing was mainly driven by the type of fibre reinforcement and not by the type of polymer matrix used, increasing further the future potential of new types of recyclable polymer matrix systems.

## Figures and Tables

**Table 1:** Average thickness and fibre volume fractions of the fabricated composite laminates [14].

Short Name	Composite Laminates	Average Thickness (mm)	Average Fibre Volume Fraction (%)	
			Flax	Glass
FFRP1	Flax/petro-epoxy	2.62 ± 0.10	47.41 ± 1.91	-
FFRP2	Flax/bio-based recyclable epoxy	2.73 ± 0.13	45.58 ± 2.24	-
FFRP3	Flax/acrylic thermoplastic	2.50 ± 0.07	49.62 ± 1.40	-
GFRP1	Glass/petro-epoxy	2.25 ± 0.08	-	58.81 ± 2.12
GFRP2	Glass/bio-based recyclable epoxy	2.21 ± 0.17	-	60.10 ± 4.41
GFRP3	Glass/acrylic thermoplastic	2.17 ± 0.14	-	60.93 ± 3.60

**Table 2:** Average moisture uptake (%) of the flexural test specimens during hygrothermal ageing.

Ageing Conditions	Ageing Period (Days)	Moisture Uptake (%)					
		FFRP1	FFRP2	FFRP3	GFRP1	GFRP2	GFRP3
23 °C	7	9.95 ± 0.71	10.01 ± 1.03	11.38 ± 1.01	0.19 ± 0.04	0.15 ± 0.03	0.14 ± 0.03
	14	12.11 ± 0.66	12.41 ± 0.84	12.82 ± 1.01	0.28 ± 0.02	0.26 ± 0.06	0.30 ± 0.13
	28	13.24 ± 1.10	13.76 ± 0.21	13.55 ± 1.71	0.38 ± 0.05	0.31 ± 0.08	0.34 ± 0.09
	56	12.64 ± 0.87	14.39 ± 0.23	12.67 ± 1.40	0.44 ± 0.02	0.33 ± 0.04	0.42 ± 0.13
40 °C	7	12.09 ± 0.69	11.42 ± 0.44	12.13 ± 0.82	0.40 ± 0.02	0.28 ± 0.05	0.31 ± 0.13
	14	12.91 ± 1.13	13.84 ± 0.31	13.03 ± 1.69	0.60 ± 0.03	0.42 ± 0.09	0.53 ± 0.18
	28	12.44 ± 1.09	15.16 ± 0.68	13.58 ± 1.68	0.73 ± 0.06	0.67 ± 0.13	0.67 ± 0.18
	56	11.94 ± 1.07	16.19 ± 0.42	13.14 ± 1.23	0.69 ± 0.08	0.57 ± 0.17	0.57 ± 0.16
60 °C	7	11.58 ± 0.89	12.25 ± 0.15	13.73 ± 0.54	0.80 ± 0.04	0.71 ± 0.10	0.95 ± 0.13
	14	11.40 ± 0.84	13.33 ± 0.23	14.77 ± 0.43	1.01 ± 0.03	1.09 ± 0.18	1.54 ± 0.18
	28	12.14 ± 1.18	14.24 ± 0.16	14.98 ± 0.99	1.56 ± 0.14	1.40 ± 0.05	1.98 ± 0.05
	56	10.42 ± 1.15	15.52 ± 0.29	14.07 ± 1.19	2.12 ± 0.08	1.76 ± 0.29	2.31 ± 0.29

**Table 3:** Average flexural properties of the unaged and aged flax fibre composites.

Ageing Conditions	Ageing Period (Days)	Flexural Strength (MPa)			Flexural Modulus (GPa)			Flexural Strain (%)		
		FFRP1	FFRP2	FFRP3	FFRP1	FFRP2	FFRP3	FFRP1	FFRP2	FFRP3
Unaged	0	261.83 ± 1.18	277.56 ± 5.77	220.35 ± 4.19	26.65 ± 0.46	26.45 ± 0.74	24.89 ± 2.26	1.78 ± 0.03	1.76 ± 0.11	1.85 ± 0.09
23 °C	7	91.99 ± 2.72	95.88 ± 4.24	82.06 ± 6.52	8.27 ± 0.42	9.47 ± 0.23	7.96 ± 0.62	3.64 ± 0.14	3.62 ± 0.22	3.39 ± 0.28
	14	86.84 ± 2.48	87.87 ± 3.87	79.09 ± 4.04	7.65 ± 0.10	8.76 ± 0.31	7.53 ± 0.52	3.71 ± 0.08	3.63 ± 0.30	3.56 ± 0.11
	28	90.36 ± 3.23	91.20 ± 3.77	77.19 ± 6.07	7.97 ± 0.51	8.59 ± 0.80	7.30 ± 0.94	3.60 ± 0.10	3.53 ± 0.10	3.31 ± 0.22
	56	87.16 ± 3.12	92.13 ± 1.86	84.33 ± 6.74	7.39 ± 0.65	9.03 ± 0.29	7.59 ± 0.59	3.84 ± 0.13	3.49 ± 0.18	3.59 ± 0.16
40 °C	7	84.52 ± 2.66	92.21 ± 3.67	76.69 ± 6.33	6.70 ± 0.29	8.41 ± 0.84	6.94 ± 0.58	3.96 ± 0.17	3.77 ± 0.18	3.58 ± 0.30
	14	87.01 ± 3.66	89.35 ± 3.13	71.85 ± 10.28	6.48 ± 0.29	7.59 ± 0.31	6.26 ± 0.78	4.14 ± 0.13	3.98 ± 0.20	3.52 ± 0.28
	28	86.51 ± 5.23	91.94 ± 5.81	76.25 ± 10.70	6.44 ± 0.37	7.95 ± 1.40	6.69 ± 0.97	4.02 ± 0.28	3.94 ± 0.46	3.61 ± 0.26
	56	86.11 ± 5.76	86.62 ± 5.09	79.00 ± 7.08	6.37 ± 0.24	7.48 ± 0.44	7.04 ± 0.80	4.12 ± 0.27	3.89 ± 0.27	3.69 ± 0.51
60 °C	7	81.34 ± 4.08	77.54 ± 3.84	60.87 ± 5.64	5.64 ± 0.32	5.41 ± 0.28	4.13 ± 0.34	4.31 ± 0.20	4.29 ± 0.25	4.04 ± 0.20
	14	83.08 ± 3.22	75.01 ± 2.01	56.53 ± 3.18	5.19 ± 0.44	5.11 ± 0.19	4.03 ± 0.32	4.41 ± 0.26	4.26 ± 0.22	4.00 ± 0.05
	28	84.07 ± 3.76	78.08 ± 1.44	61.93 ± 5.60	5.52 ± 0.31	5.67 ± 0.44	4.25 ± 0.61	4.24 ± 0.22	4.20 ± 0.25	4.06 ± 0.19
	56	81.97 ± 5.44	73.86 ± 3.23	60.84 ± 3.25	4.96 ± 0.17	4.95 ± 0.44	4.14 ± 0.45	4.33 ± 0.18	4.20 ± 0.27	4.09 ± 0.38

**Table 4:** Average flexural properties of the unaged and aged glass fibre composites.

Ageing Conditions	Ageing Period (Days)	Flexural Strength (MPa)			Flexural Modulus (GPa)			Flexural Strain (%)		
		GFRP1	GFRP2	GFRP3	GFRP1	GFRP2	GFRP3	GFRP1	GFRP2	GFRP3
Unaged	0	876.92 ± 53.33	989.89 ± 27.80	1016.49 ± 33.64	44.07 ± 2.43	46.02 ± 1.84	46.17 ± 1.42	2.23 ± 0.30	2.41 ± 0.13	2.48 ± 0.08
23 °C	7	725.83 ± 79.73	917.69 ± 17.11	928.66 ± 29.23	44.67 ± 2.23	40.75 ± 3.44	44.94 ± 1.35	1.74 ± 0.28	2.55 ± 0.23	2.24 ± 0.10
	14	674.78 ± 54.38	881.24 ± 29.40	890.51 ± 35.14	43.16 ± 2.87	43.94 ± 0.74	44.68 ± 1.48	1.74 ± 0.34	2.17 ± 0.04	2.15 ± 0.14
	28	726.40 ± 36.86	878.93 ± 72.57	891.79 ± 37.37	39.91 ± 0.60	42.71 ± 2.00	45.09 ± 1.18	1.92 ± 0.12	2.28 ± 0.09	2.18 ± 0.10
	56	693.86 ± 62.72	866.52 ± 43.70	908.55 ± 31.78	43.34 ± 2.75	44.55 ± 0.70	46.06 ± 0.68	1.70 ± 0.23	2.13 ± 0.08	2.15 ± 0.12
40 °C	7	725.05 ± 74.21	910.95 ± 13.08	897.84 ± 42.29	41.48 ± 1.30	44.58 ± 1.40	44.54 ± 1.20	1.86 ± 0.23	2.23 ± 0.04	2.19 ± 0.15
	14	634.78 ± 119.37	872.20 ± 53.56	830.38 ± 53.66	43.71 ± 2.59	43.36 ± 1.85	44.77 ± 0.70	1.53 ± 0.34	2.18 ± 0.05	1.97 ± 0.18
	28	665.18 ± 89.43	845.87 ± 44.23	856.78 ± 49.84	43.07 ± 2.97	44.24 ± 0.77	44.84 ± 1.44	1.62 ± 0.18	2.08 ± 0.15	2.07 ± 0.12
	56	598.26 ± 36.34	687.13 ± 115.80	873.71 ± 28.70	41.15 ± 1.06	43.42 ± 1.08	45.35 ± 1.09	1.51 ± 0.15	1.65 ± 0.32	2.06 ± 0.12
60 °C	7	625.06 ± 79.63	796.37 ± 54.08	723.89 ± 23.75	42.31 ± 3.03	44.57 ± 2.16	43.98 ± 1.59	1.55 ± 0.18	1.90 ± 0.07	1.87 ± 0.29
	14	594.49 ± 95.28	730.09 ± 44.03	620.50 ± 77.74	43.61 ± 1.51	43.43 ± 1.03	42.61 ± 1.81	1.43 ± 0.27	1.75 ± 0.12	1.53 ± 0.19
	28	455.33 ± 85.23	711.50 ± 60.06	526.40 ± 41.17	37.58 ± 3.42	43.91 ± 1.15	43.09 ± 2.06	1.31 ± 0.13	1.68 ± 0.12	1.25 ± 0.08
	56	344.88 ± 27.84	654.54 ± 62.74	509.45 ± 29.75	37.57 ± 2.68	41.88 ± 1.04	42.83 ± 1.65	0.99 ± 0.05	1.62 ± 0.16	1.22 ± 0.06

**Table 5:** Average moisture uptake (%) of the DMA test specimens during hygrothermal ageing.

Ageing Conditions	Ageing Period (Days)	Moisture Uptake (%)					
		FFRP1	FFRP2	FFRP3	GFRP1	GFRP2	GFRP3
23 °C	7	9.66 ± 0.18	8.77 ± 0.83	11.13 ± 0.73	0.19 ± 0.01	0.21 ± 0.02	0.18 ± 0.03
	14	11.36 ± 0.20	9.85 ± 0.74	11.44 ± 0.75	0.24 ± 0.04	0.19 ± 0.01	0.28 ± 0.02
	28	13.31 ± 0.81	10.95 ± 0.68	12.22 ± 0.54	0.43 ± 0.06	0.31 ± 0.03	0.34 ± 0.03
	56	12.32 ± 0.66	11.68 ± 0.68	10.53 ± 0.82	0.54 ± 0.07	0.41 ± 0.03	0.44 ± 0.01
40 °C	7	12.67 ± 0.36	10.93 ± 0.69	11.98 ± 0.77	0.51 ± 0.06	0.36 ± 0.03	0.37 ± 0.04
	14	13.11 ± 0.36	11.95 ± 0.83	12.35 ± 0.31	0.70 ± 0.06	0.56 ± 0.04	0.47 ± 0.03
	28	12.80 ± 0.45	13.50 ± 0.64	13.13 ± 0.40	0.84 ± 0.19	0.73 ± 0.07	0.57 ± 0.04
	56	11.84 ± 0.39	13.30 ± 0.62	10.73 ± 0.20	0.81 ± 0.16	1.04 ± 0.06	0.54 ± 0.02
60 °C	7	12.38 ± 0.77	13.28 ± 0.50	14.64 ± 0.41	0.79 ± 0.03	0.82 ± 0.08	1.05 ± 0.04
	14	11.87 ± 0.54	13.85 ± 0.59	14.00 ± 0.89	1.04 ± 0.13	1.11 ± 0.05	1.57 ± 0.08
	28	11.94 ± 0.33	15.21 ± 1.15	15.22 ± 0.91	1.66 ± 0.27	1.51 ± 0.11	2.25 ± 0.19
	56	11.25 ± 0.34	15.68 ± 0.72	12.79 ± 0.96	2.47 ± 0.24	2.77 ± 0.30	2.46 ± 0.31

**Table 6:** Average storage modulus (E’) of the unaged and aged composite samples.

Ageing Conditions	Ageing Period (Days)	Storage Modulus, E’ (MPa)					
		FFRP1	FFRP2	FFRP3	GFRP1	GFRP2	GFRP3
Unaged	0	21.99 ± 2.19	20.96 ± 0.52	19.24 ± 1.55	37.84 ± 4.55	34.02 ± 1.72	33.44 ± 5.61
23 °C	7	10.42 ± 1.60	10.80 ± 1.34	13.05 ± 1.84	38.24 ± 1.77	33.59 ± 2.38	36.88 ± 1.48
	14	10.30 ± 1.94	10.42 ± 1.99	12.21 ± 1.50	37.27 ± 2.86	33.74 ± 1.43	36.23 ± 1.05
	28	11.50 ± 1.89	11.15 ± 2.28	12.00 ± 1.32	34.39 ± 5.95	33.48 ± 1.91	37.11 ± 1.08
	56	8.74 ± 1.25	9.38 ± 1.41	10.76 ± 1.21	36.05 ± 4.14	33.31 ± 1.46	36.15 ± 0.36
40 °C	7	8.07 ± 0.70	10.01 ± 2.00	9.60 ± 1.78	36.39 ± 3.76	34.29 ± 1.20	37.62 ± 1.66
	14	9.11 ± 1.15	8.69 ± 0.96	8.52 ± 2.40	32.84 ± 4.13	32.99 ± 0.82	36.20 ± 1.72
	28	9.47 ± 1.14	8.49 ± 1.83	10.21 ± 1.67	35.83 ± 5.56	32.47 ± 2.27	36.70 ± 0.89
	56	8.87 ± 1.22	7.68 ± 1.58	9.06 ± 2.21	37.00 ± 3.60	33.92 ± 0.93	36.60 ± 1.96
60 °C	7	6.54 ± 0.72	6.23 ± 0.94	5.33 ± 0.25	38.00 ± 1.09	32.87 ± 1.52	27.53 ± 2.39
	14	6.34 ± 0.57	5.97 ± 1.00	5.69 ± 0.57	37.03 ± 1.20	31.21 ± 1.15	25.03 ± 1.84
	28	6.37 ± 0.81	6.08 ± 0.33	6.62 ± 0.94	29.99 ± 4.12	31.37 ± 1.54	28.96 ± 3.02
	56	6.26 ± 0.65	5.49 ± 0.56	5.45 ± 0.35	22.92 ± 0.73	30.84 ± 1.47	28.48 ± 1.82

**Table 7:** Average glass transition temperature (T_g_) of the unaged and aged composite samples as a function of hygrothermal ageing under different conditions.

Ageing Conditions	Ageing Period (Days)	Glass Transition Temperature, T_g_ (°C)					
		FFRP1	FFRP2	FFRP3	GFRP1	GFRP2	GFRP3
Unaged	0	75.53 ± 1.20	90.19 ± 1.33	101.12 ± 0.43	69.94 ± 0.49	91.18 ± 1.05	95.08 ± 0.84
23 °C	7	69.33 ± 2.29	74.66 ± 0.29	99.61 ± 2.31	64.61 ± 0.31	89.23 ± 1.42	88.99 ± 0.40
	14	70.33 ± 1.32	75.16 ± 1.16	100.31 ± 2.03	64.42 ± 0.94	89.72 ± 0.54	87.25 ± 0.78
	28	75.61 ± 1.79	75.86 ± 1.76	100.86 ± 1.99	62.92 ± 1.16	88.60 ± 2.24	87.48 ± 1.54
	56	79.83 ± 1.68	75.58 ± 0.21	101.93 ± 1.32	59.73 ± 1.04	87.79 ± 0.88	83.93 ± 0.35
40 °C	7	78.52 ± 1.86	75.87 ± 0.25	105.10 ± 1.36	59.85 ± 0.79	87.74 ± 0.30	84.02 ± 1.13
	14	83.02 ± 1.06	77.05 ± 0.27	104.84 ± 2.36	58.41 ± 0.64	85.83 ± 1.40	82.22 ± 1.35
	28	88.57 ± 2.68	78.69 ± 2.27	109.19 ± 0.62	59.18 ± 2.66	81.22 ± 1.28	82.98 ± 3.70
	56	94.49 ± 1.00	79.72 ± 0.87	109.34 ± 1.66	58.44 ± 0.81	75.39 ± 0.78	83.74 ± 2.99
60 °C	7	95.90 ± 2.94	83.37 ± 1.83	113.81 ± 1.41	57.83 ± 1.13	79.98 ± 0.71	79.41 ± 0.27
	14	101.07 ± 1.89	86.67 ± 6.12	114.97 ± 1.62	58.32 ± 0.14	76.56 ± 0.82	80.81 ± 6.16
	28	109.55 ± 7.41	90.17 ± 7.00	116.08 ± 0.38	62.57 ± 3.01	72.98 ± 1.60	83.10 ± 6.70
	56	106.78 ± 0.71	86.08 ± 2.92	119.21 ± 0.67	67.84 ± 1.29	63.25 ± 1.13	81.99 ± 15.90

## Data Availability

Not applicable.
